# Mid-regional pro-adrenomedullin (MR-proADM), a marker of positive fluid balance in critically ill patients: results of the ENVOL study

**DOI:** 10.1186/s13054-016-1540-x

**Published:** 2016-11-09

**Authors:** Bernard Vigué, Pierre-Etienne Leblanc, Frédérique Moati, Eric Pussard, Hussam Foufa, Aurore Rodrigues, Samy Figueiredo, Anatole Harrois, Jean-Xavier Mazoit, Homa Rafi, Jacques Duranteau

**Affiliations:** 1 Département d’Anesthésie-Réanimation, Hôpital de Bicêtre, Université Paris-Sud, Hôpitaux Universitaires Paris-Sud, Assistance Publique-Hôpitaux de Paris, Le Kremlin Bicêtre, Paris, France; 2 Service de biophysique et de médecine nucléaire, Centre Hospitalier Universitaire de Bicêtre, Assistance publique – Hôpitaux de Paris, Paris, France; 3 Service de Génétique Moléculaire, Pharmacogénétique et Hormonologie, Inserm U1185, Centre Hospitalier Universitaire de Bicêtre, Assistance publique – Hôpitaux de Paris, Paris, France; 4 Thermo Fisher Scientific, Asnières sur Seine, France

**Keywords:** MR-proADM, Adrenomedullin, Intensive care unit, Plasma biomarker, Fluid balance, Fluid overload, Sodium overload

## Abstract

**Background:**

The optimal control of blood volume without fluid overload is a main challenge in the daily care of intensive care unit (ICU) patients. Accordingly this study focused on the identification of biomarkers to help characterize fluid overload status.

**Methods:**

Sixty-seven patients were studied from ICU admission to day 7 (D_7_). Blood and urine samples were taken daily and sodium and water balance strictly calculated resulting in a total cumulative assessment of ∆Na^+^ and ∆H_2_O. Furthermore, plasmatic biomarkers (cortisol, epinephrine, norepinephrine, renin, angiotensin II, aldosterone, pro-endothelin, copeptine, atrial natriuretic peptide, erythropoietin, mid-regional pro-adrenomedullin (MR-proADM)) and Sequential Organ Failure Assessment (SOFA) scores were measured at D_2_, D_5_ and D_7_. Blood volumes were measured with ^51^Cr fixed on red blood cells at D_2_ and D_7_.

**Results:**

The ∆Na^+^ or ∆H_2_O were increased in all patients but never related to blood volumes at D_2_ nor D_7_. Total blood volumes were at normal values with constantly low red blood cell volumes and normal or decreased plasmatic volume. Weight, plasmatic proteins, and hemoglobin were weakly related to ∆Na^+^ or ∆H_2_O. Amongst all tested biomarkers, only MR-proADM was related to sodium and fluid overload. This biomarker was also a predictor of SOFA scores.

**Conclusions:**

Plasmatic concentration in MR-proADM seems to be a good surrogate for evaluation of ∆Na^+^ or ∆H_2_O and predicts sodium and extracellular fluid overload.

**Trial registration:**

ClinicalTrials.gov: NCT01858675 in May 13, 2013.

**Electronic supplementary material:**

The online version of this article (doi:10.1186/s13054-016-1540-x) contains supplementary material, which is available to authorized users.

## Background

The maintenance of optimal blood volume without the development of a positive fluid balance is a major challenge in the daily care of patients suffering from acute traumatic, subarachnoid hemorrhage or infectious inflammatory disorders [1–10]. Indeed, capillary leak significantly contributes to the development of tissue edema and causes persistent hypovolemia despite fluid resuscitation [11–13]. The consequence is two-fold, with (1) a large volume fluid resuscitation and (2) an increase in tissue edema with impairment of microcirculation architecture and oxygen diffusion. This fluid overload caused by fluid resuscitation and excess sodium can be the source of organ dysfunction, including acute lung injury, abdominal compartment syndrome and acute renal injury [2–5, 14, 15], thereby contributing to higher mortality [7–9]. A 4-kg weight gain, corresponding to an accumulation of 4 L of water and 36 g of sodium chloride, is the limit beyond which morbidity and mortality increase [3, 16–18].

The accurate monitoring of fluid balance is therefore crucial in guiding fluid resuscitation. However, there is no gold standard method for complete measurement and the use of input/output charts in intensive care unit (ICU) patients is notorious for being incomplete and inaccurate. Consequently, accurate and reproducible methods to improve the monitoring of fluid balance are essential.

Several plasmatic biomarkers may contribute to arbitrating conflict between the control of optimal blood volume and the development of fluid overload: these include stress hormones, such as cortisol and catecholamines; hormones involved in volume regulation by sodium chloride or water retention, such as the renin-angiotensin II-aldosterone system [19]; vasopressin, expressed by (CT)-pro-arginine vasopressin, known as copeptin [20]; endothelin expressed by pro-endothelin and atrial natriuretic peptide (ANP), measured by pro-ANP; factors involved in the production of red blood cells, such as erythropoietin (EPO) [21]; and factors that repair the endothelium after injury, such as mid-regional pro-adrenomedullin (MR-proADM) [22, 23].

The first aim of the *Etude des marqueurs iNnovants de la VOLémie* (ENVOL study) was to identify a reliable surrogate biomarker capable of predicting blood volumes and/or cumulative sodium and water balance (∆Na^+^ and ∆H_2_O). We also tried to evaluate any relationship between extracellular volume and blood volume measurements. For these purposes, we precisely characterized changes in extracellular and blood volumes and changes in several plasmatic biomarkers involved in volume regulation during the first 7 days of ICU stay.

## Methods

This prospective, 7-day observational study was conducted between March 2012 and September 2014 in the 38-bed Department of Anesthesiology and Intensive Care at Bicêtre University Hospital, in Le Kremlin-Bicêtre, France. The Institutional Review Board of the hospital (*Comité de protection des personnes Ile de France* VII) approved the study on December 2011 (reference 11-045). Written informed consent was systematically obtained from all participants included in the study or from a relative, in accordance with French legal ethics.

We studied four groups of ICU patients, including those with severe brain trauma (SBT), aneurysmal subarachnoid hemorrhage (SAH), severe non-cerebral trauma (NCT) or postoperative peritonitis with septic shock (PPS). Patients were included if they required continuous mechanical ventilation on day 2. SBT was defined as brain trauma with a Glasgow coma score of <9. Patients with SAH were included in the presence of a score ≥4 on the World Federation of Neurosurgical Societies (WFNS) scale [24]. Patients with NCT were included when the Injury Severity Score was ≥25. Patients with PPS were included after abdominal surgery complicated by manifestations of hemodynamic shock, including hypotension and low cardiac output or lactate concentration >4 mmol/L. Patients <18 years, pregnant or presenting with New York Heart Association (NYHA) graded ≥ II were excluded.

### Data collection

During the ICU stay, general and demographic data were collected, including age, sex, weight, height, simplified index of illness severity after the first 48 h (Simplified Index of Gravity (IGS) II) and admission date. Sequential Organ Failure Assessment (SOFA) scores were measured upon admission (day 0 (D_0_)), and on days 2, 5 and 7 (D_2_, D_5_ and D_7_) [25]. Mean arterial pressure, doses of norepinephrine and core temperature were recorded daily. Blood laboratory tests included measurements of hemoglobin, proteins, electrolytes, creatinine and urinary concentrations of Na^+^, K^+^, Cl^-^, urea, creatinine and osmolarity. The urinary electrolytes, creatinine, urea and osmolarity were measured in the morning and the total 24-h output was used to calculate the previous day’s loss of Na^+^, K^+^, urea and the creatinine clearance. Weight was carefully measured using weighing beds and recorded on D_2_ and D_7_. The baseline weight was that recorded by the patient or a relative.

The daily intravenous fluid administration was based on the monitoring of heart rate, arterial pressure, blood lactate concentration, serial echocardiograms, cardiac filling pressures and output and signs of fluid responsiveness in ventilated patients [26, 27].

### Evaluation of extracellular volume

Sodium and fluid balances were calculated daily in order to estimate changes in extracellular space. The previous day’s inputs and outputs of sodium and water were calculated each morning. All other losses were measured, including from ileostomies and external ventricular drainage when present. Sodium losses were measured from all liquids and deducted from sodium intake. The difference between water administration from enteral nutrition and daily crystalloids or colloid infusion and water loss was calculated. Insensitive losses were adjusted for body temperature. The sodium and water gains or losses were calculated daily and added to the previous day’s measurements as cumulative fluid balance (∆Na^+^ and ∆H_2_O). A >36 g ∆Na^+^ or >4 L ∆H_2_O was defined as fluid overload [16–18]. The creatinine clearance was calculated daily. All calculations were made by one caregiver and verified by another (PEL, HF and BV).

### Blood volume measurements

The red blood cell volume (RBCV) was measured on D_2_ and D_7_ (±1 day), using 10 mL of the patient’s red blood cells (RBC) labeled with radioactive chromium (^51^Cr-RBC). We re-injected a known quantity of radioactive RBC intravenously and collected two arterial samples 10 and 30 minutes later. From the radioactivity of these samples we derived RBCV in mL/kg using the patient’s body weight recorded before admission [28]. The arterial hematocrit and RBCV defined the total blood volume (TBV), in mL/kg and the plasma volume (PV), in mL/kg. The normal values are 32 ± 6 mL/kg for RBCV, 72 ± 14 mL/kg for TBV, and 40 ± 8 mL/kg for PV. Hypovolemia was noted when TBV was <20 % of normal values [28]. On D_7_, we also measured PV by intravenously injecting a small amount of albumin labeled with radioactive iodine (^125^I-albumin), and collected arterial samples at 10 and 30 minutes and at 2 h [29]. The normal PV measured with ^125^I is 45 ± 10 mL/kg, slightly larger than that measured with ^51^Cr-RBC [28].

### Biomarker analysis

Plasma biomarkers were analyzed on D_2_, D_5_ and D_7_. MR-proADM, Pro-ANP, renin, angiotensin II, aldosterone, cortisol, norepinephrine and epinephrine, copeptin, pro-endothelin and EPO were measured for potential interference with extracellular or blood volumes. All biologic biomarkers were analyzed together after inclusion in November 2014.

### Statistical analysis

Because our original aim was to assess the correlation of the biomarkers with intravascular volumes, the study sample size was calculated considering that brain natriuretic peptide (BNP) and EPO are good surrogate markers of intravascular volumes. We then used published values of BNP and EPO concentrations in similar patients [21, 30] to calculate the number of patients needed (three groups of patients with hypervolemia, normovolemia and hypovolemia, respectively, considering the initial protocol with a 50 % between-group difference and power of 80 %). Data were analyzed using R [31]. The normality of data distribution was verified using quantile-quantile plots and Shapiro’s test. Because most data were measured with error, Deming regression with equal variances was used to calculate the slope of the regression curves.

Because biomarkers were approximately log-normally distributed, comparisons between D_2_, D_5_ and D_7_ values were performed on the log transformed data using the Tukey test after analysis of variance (ANOVA). As our objective was also the measurement of extracellular volume, we also studied fluid overload. For this purpose we considered 4 L and 36 g as ∆H_2_O and ∆Na^+^ (equivalent to 4 L of saline), respectively, for the cutoff of response variables [16–18].

We also calculated the performance of biomarkers to predict a SOFA score >9. The calculation of ∆H_2_O and ∆Na^+^ and SOFA score were performed on the same day as the biomarkers were assessed. Because of the large number of variables that were candidates for inclusion in multivariate analysis, we first used random forest regression [32] to select the most pertinent demographic and biological covariates explaining fluid and sodium overload and SOFA score. This was followed by linear mixed effect regression in which subject and day of measurement were considered as random effects due to correlation between days of measurement.

Receiver operating characteristic (ROC) curves were constructed to calculate the performance of the biomarkers in predicting fluid and sodium overload (∆H_2_O and ∆Na^+^) and SOFA score. The optimal sensitivity/specificity cutoff for predictive variables was calculated, using the non-weighted Youden index. The statistical significance was set at *P* <0.05. Data are reported as means ± standard deviation (SD), medians (25–75 percentiles) or counts and percentages or 95 % confidence interval (95 % CI).

## Results

In the first 7 days after admission to the ICU, 67 patients including those with SBT (n = 21), SAH (n = 20), NCT (n = 20) and PPS (n = 6) were studied. General demographic data, the number of patients studied in each group and SOFA scores on D_0_, D_2_, D_5_ and D_7_ are shown in Table 1.

**Table 1:** General and demographic characteristics and SOFA scores in the entire study sample and in each study group

	All patients *n* = 67	Severe brain trauma *n* = 21	Subarachnoid hemorrhage *n* = 20	Non-cerebral trauma *n* = 20	Peritonitis with shock *n* = 6
Age, years	46 ± 19	38 ± 16	53 ± 14	39 ± 18	69 ± 16
Women/men	24/43	18/3	11/9	6/14	4/2
Weight, kg	75 ± 18	73 ± 14	53 ± 14	84 ± 22	71 ± 9
Height, cm	172 ± 10	178 ± 9	169 ± 9	173 ± 11	161 ± 8
IGS II	43 ± 13	49 ± 9	42 ± 12	37 ± 11	54 ± 10
Days in ICU	27 ± 22	34 ± 3	27 ± 16	22 ± 13	21 ± 6
SOFA scores					
D_0_	10 (9–13)	10 (9–11)	9 (7–10)	12 (10–14)	15 (14–16)
D_2_	8 (6–11)	7 (6–11)	8 (4–9)	8 (8–11)	14 (12–14)
D_5_	5 (3–8)	5 (3–7)	4 (3-7)	5 (3-8)	7 (3–11)
D_7_	4 (2–7)	5 (2–8)	4 (3–6)	3 (1–6)	4 (1–7)

Values are means ± SD or median (interquartile 25–75 range). *IGS II* Index Gravity Score, *SOFA* Sequential Organ Failure Assessment

### Sodium and hydric balance

**Fig. 1:**
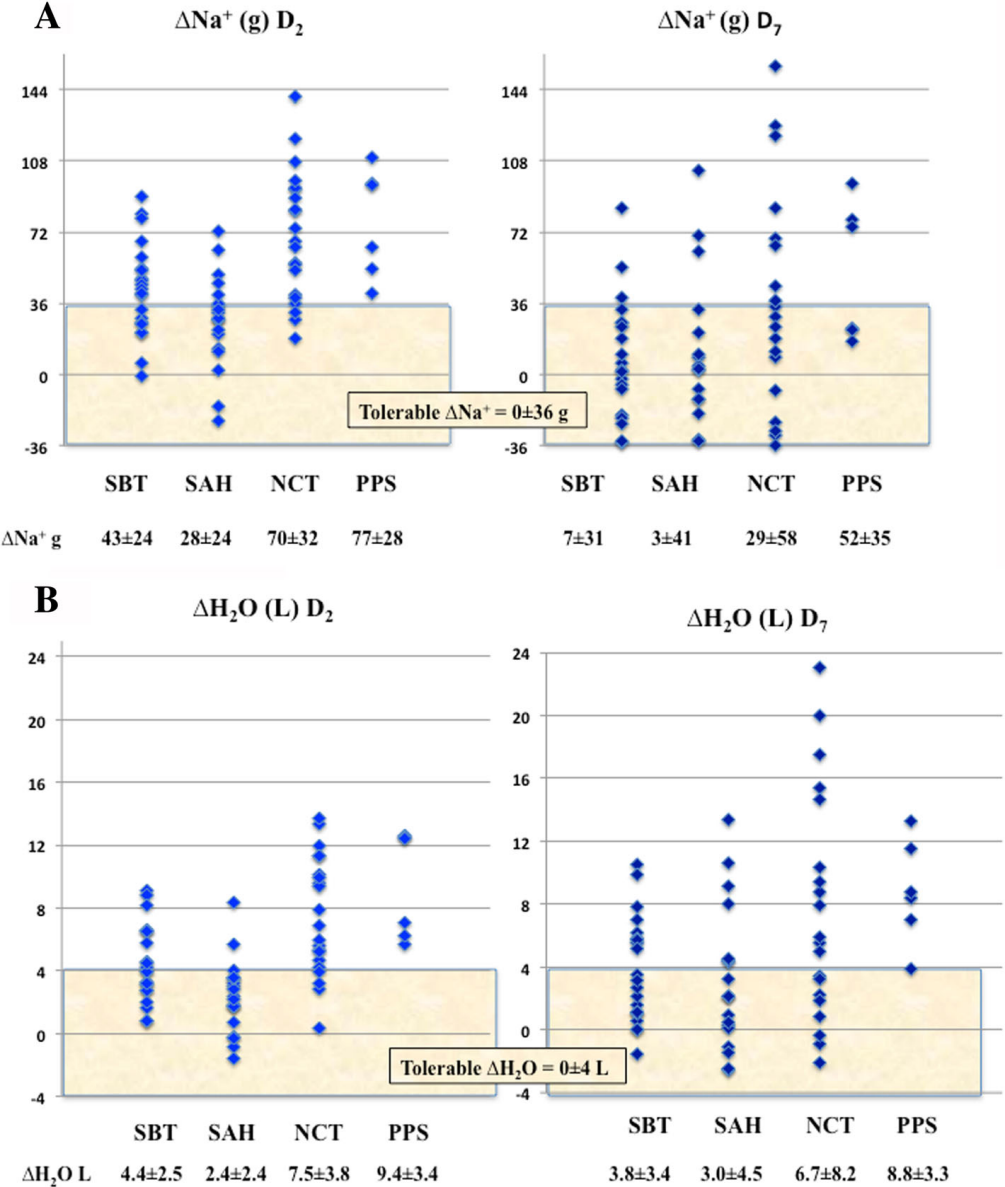
**a** Cumulative sodium balance (∆Na^+^, g) on day 2 (*D*_*2*_) and *D*_*7*_ in the 67 patients studied. **b** Cumulative fluid balance (∆H_2_O, L) on *D*_*2*_ and *D*_*7*_. Thresholds of 36 g for sodium and 4 L for water, corresponding to 4 L of saline and described as a *tolerable* fluid overload, are marked as *shaded zone* around equilibrate balances. *SBT* severe brain trauma, *SAH* aneurysmal subarachnoid hemorrhage, *NCT* severe non-cerebral trauma, *PPS* postoperative peritonitis with shock

The cumulative sodium and water gains are reported as ∆Na^+^ and ∆H_2_O on D_2_ and D_7_ in each study group (Fig. 1). A fluid overload (i.e. more than 36 g of Na^+^ or 4 L of H_2_O) on D_2_ was observed in 43 patients (64 %) for ∆Na^+^ and 36 for ∆H_2_O (54 %), and on D_7_ in 18 (27 %) for ∆Na^+^ and 33 (49 %) for ∆H_2_O. This sodium and water positive balances on D_2_ and D_7_ were observed in all groups, with a higher increase in sodium and water in the NCT and PPS than in the SBT and SAH groups. For example, on D_2_, a cumulative increase in Na^+^ of 70 ± 32 and 77 ± 28 g was measured in the NCT and PPS group, in contrast to 43 ± 24 and 28 ± 24 g in the SBT and SAH groups (*P* < 0.0001, Fig. 1a). The ∆Na^+^ was related to ∆H_2_O, confirming that retained water is related to retained sodium (∆Na^+^(g) = 7.2∆H_2_O(L)-4.0; *r* ^2^ = 0.67; *P* < 0.0001). As a known indicator of extracellular space, plasma concentration of proteins and variation in weight were related to ∆Na^+^ and ∆H_2_O, though these relationships were weak (*r* ^2^ = 0.44 and *r* ^2^ = 0.35 for plasma proteins and ∆Na^+^ or ∆H_2_O, *r* ^2^ = 0.27 and *r* ^2^ = 0.33 for ∆ weight and ∆Na^+^ or ∆H_2_O, *P* < 0.001 for all). Like plasma proteins, hemoglobin was weakly related to ∆Na^+^ (*r* ^2^ = 0.15) or ∆H_2_0 (*r* ^2^ = 0.23), *P* < 0.001.

### Blood volumes

**Fig. 2:**
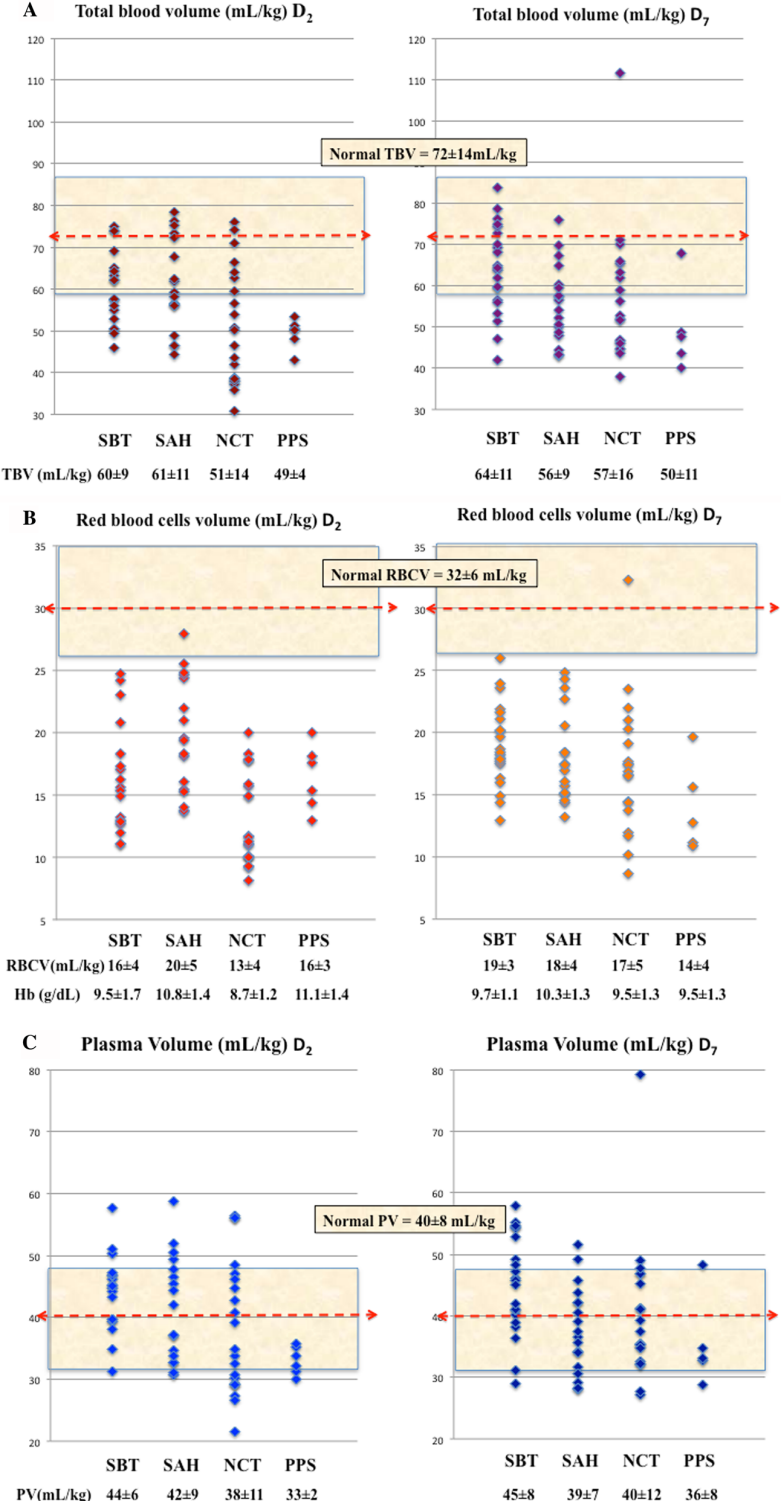
Total blood volume (*TBV*, mL/Kg) (**a**), red blood cell volume (*RBCV*, mL/Kg) (**b**) and plasmatic volume (*PV*, mL/Kg) (**c**) on day 2 (*D*_*2*_) and *D*_*7*_. Thresholds of 20 % around normal values are marked as *shaded zones. SBT* severe brain trauma, *SAH* aneurysmal subarachnoid hemorrhage, *NCT* severe non-cerebral trauma, *PPS* postoperative peritonitis with shock

TBV, RBCV and PV were measured with ^51^Cr in 62 patients on D_2_ and in 63 patients on D_7_ (with ^125^I-albumin in 58 patients on D_7_). TBV, RBCV and PV are shown in Fig. 2. A decrease in TBV was observed in most patients. Only 28 patients on D_2_ (45 %) and 31 on D_7_ (49 %) were in the normal range. Hypovolemia (TBV <20 %) was present in 34 patients (55 %) on D_2_ and 32 patients (51 %) on D_7_. Low RBCV was observed in all but three transfused patients (Fig. 2b). Decrease in RBCV was sometimes notably low with a RBCV <50 % in 34 patients (55 %) on D_2_ and 21 patients (33 %) on D_7_. We found no significant relationship between TBV or RBCV and ∆Na^+^ or ∆H_2_O (see Additional file 1).

The distribution of PV was in the normal range (Fig. 2c). Only 13 patients on D_2_ (21 %) and 10 patients on D_7_ (16 %) were below the 20 % range. Importantly, we found no relationship between PV and ∆Na^+^ or ∆H_2_O (see Additional file 1). Identically, no correlation was observed between PV and plasma proteins. Hemoglobin concentration was weakly related to RBCV (*r* ^2^ = 0.33, *P* < 0.001) and was not related to PV (*r* ^2^ = 0.026, *P* = 0.07). On D_7_, PV was measured by ^125^I-albumin in 58 patients. There was a statistically significant relationship between PV calculated from ^51^Cr-RBC and PV directly measured with ^125^I-albumin (*P* < 0.0001). The slope of the Deming regression was 0.852 (0.610–1.08) with an *r* ^2^ value of 0.75.

### Biomarkers

Detailed kinetics of all biomarkers are shown in Table 2. Most biomarkers increased on D_2_ and decreased significantly on D_5_ and D_7_ (copeptin, angiotensin II and renin). MR-proADM and EPO decreased significantly on D_7_. Cortisol, aldosterone, pro-ANP and pro-endothelin remained unchanged. Plasma norepinephrine concentration was not reliable because it was infused as a treatment.

**Table 2:** Evolution of all biomarkers

Biomarkers	Normal values (min-max)	Day 2		Day 5		Day 7	
		Number	M (25–75)	Number	M (25–75)	Number	M (25–75)
Angiotensin II (pmol/L)	19–38	58	21 (8–38)	65	9 (6–18)*	67	9 (5–12)**
Renin (pg/mL)	3–16	67	52 (14–93)	65	15 (6–64)*	67	14 (6–36)**
Aldosterone (pg/mL)	42–201	67	33 (14–111)	65	47 (19–105)	67	54 (16–127)
Pro-ANP (pmol/L)	<85	67	54 (31–110)	65	68 (48–105)	67	59 (34–97)
Pro-endothelin (pmol/L)	34–55	67	62 (47–83)	65	59 (50–70)	67	53 (43–73)
CT-proAVP (pmol/L)	1.1–16.4	67	19 (11–43)	65	13 (6–22)*	67	11 (5–21)*
MR-proADM (nmol/L)	<0.39	67	1.05 (0.79–1.85)	65	1.05 (0.75–1.46)	67	0.76 (0.6–1.15)*^¥^
Cortisol (ng/dL)	9–22	67	19 (14–27)	64	22 (18–27)	67	23 (19–28)
Epinephrin (pg/mL)	<80	66	89 (50–152)	65	95 (60–184)	67	113(55–180)
Norepinephrine (pg/mL)^a^	<450	67	1773 (630–4100)	65	762 (448–1473)	67	705 (437–1326)
EPO (mUI/mL)	6.4–63.8	66	52 (27–90)	65	38 (22–65)	67	28 (17–38)**^¥^

Min-max are the lowest and highest normal values. M (25–75) is median (interquartile 25–75 range). ^a^No statistical analysis was performed because norepinephrine was used as a treatment in a significant number of patients (*n* = 30 at day 2 (D_2_), *n* = 12 at D_5_ and *n* = 10 at D_7_). *ANP* atrial natriuretic peptide, *MR-proADM* mid-regional pro-adrenomedullin, *EPO* erythropoietin. * *P* < 0.01 vs D_2_; ** *P* < 0.001 vs D_2_; ^¥^ *P* < 0.05 vs D_5_

Of all biomarkers tested, only MR-proADM and angiotensin II were significantly related to ∆Na + (*P* = 0.01 and *P* = 0.03) and MR-proADM to ∆H_2_O (*P* < 10^-5^). SOFA was related to ∆Na^+^ (*P* < 10^-5^), MR-proADM (*P* < 10^-5^), and EPO (*P* = 0.03) (see Additional file 2). No difference was found associated to the type of pathological characteristics.

**Fig. 3:**
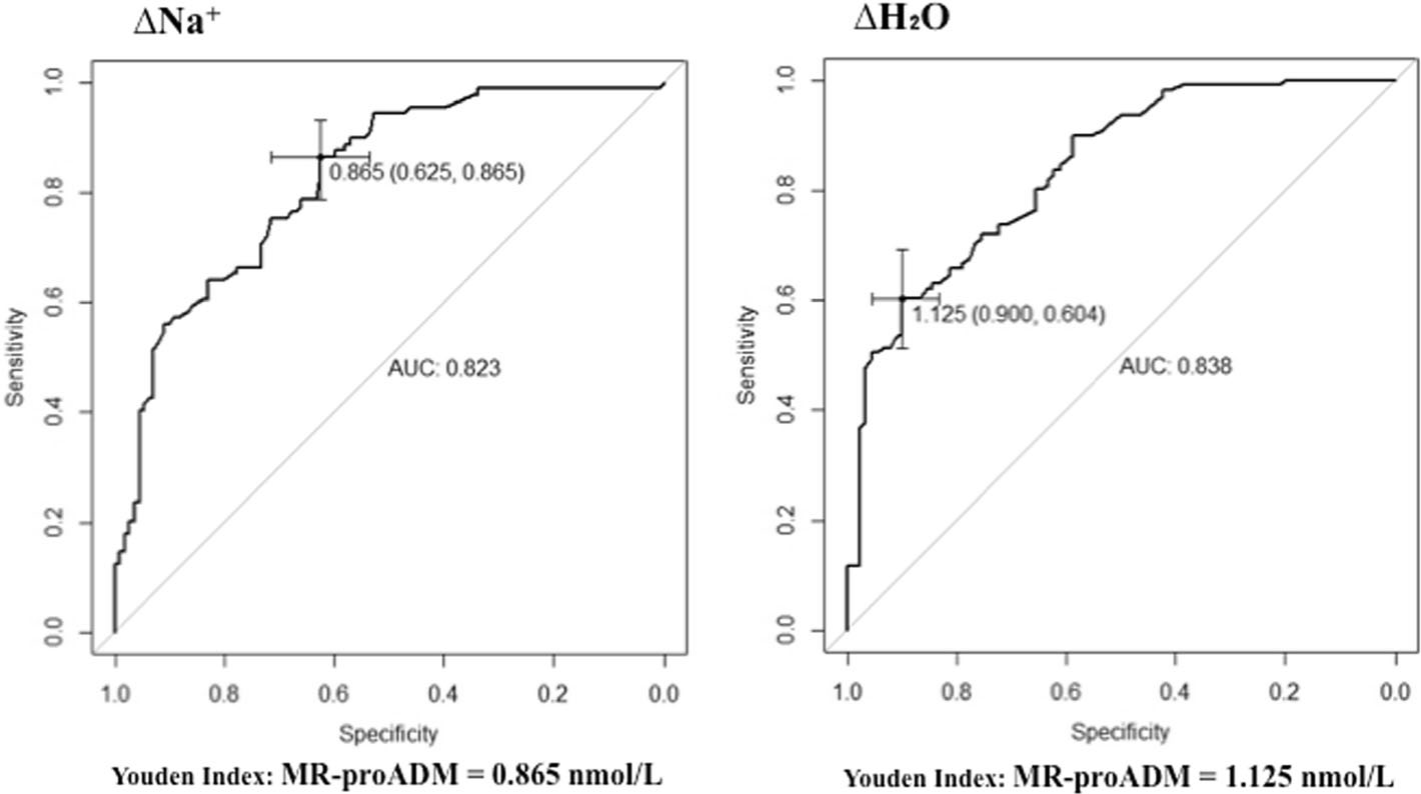
ROC curves to describe the relationship between the plasmatic concentration of mid-regional pro-adrenomedullin (*MR-proADM*) and fluid overload of 4 L for ∆H_2_O and 36 g for ∆Na^+^. Area under the curve (AUC) is 0.838 (0.780–0.888) for ∆H_2_O and 0.823 (0.764–0.880) for ∆Na^+^ determining the relationship between dangerous fluid overload and plasmatic MR-proADM. The Youden index indicates a threshold for plasmatic MR-proADM (0.865 nmol/L (specificity 0.625/sensitivity 0.865) for ∆Na^+^ >36 g and 1.125 nmol/L (specificity 0.900/sensitivity 0.604) for ∆H_2_O >4 L) to predict fluid overload

We constructed ROC curves with MR-proADM because it was the only predictor covariate common to the three variables tested (∆H_2_O, ∆Na^+^ and SOFA). MR-proADM has good discriminative properties with an area under the curve (AUC) of 0.838 (0.780–0.888) for ∆H_2_O and an AUC of 0.823 (0.764–0.880) for ∆Na^+^ (Fig. 3). We determined a threshold of MR-proADM predicting a positive balance greater than 36 g Na^+^ or 4 L H_2_O. These thresholds were 0.865 nmol/L (specificity 0.625 (0.538–0.714), sensitivity 0.865 (0.787–0.933)) for ∆Na^+^ and 1.125 nmol/L (specificity 0.900 (0.833–0.9560, sensitivity 0.604 (0.513–0.694)) for ∆H_2_O. Furthermore, MR-proADM predicted a SOFA score >9 with an AUC of 0.750 (0.658–0.830) and a threshold at 1.035 nmol/L (specificity 0.635 (0.560–0.711), sensitivity 0.762 (0.643–0.881)).

We also tested the relationships between biomarkers and measured blood volumes. It is noteworthy that among all biomarkers, only EPO was related to RBCV (*P* < 10^-5^) but the ROC curve constructed for a 50 % decrease in RBCV revealed a low AUC (0.70 (0.60–0.78)).

## Discussion

This study revealed that MR-proADM, a biomarker of endothelial permeability [22, 23], may be used as a surrogate for the increase in sodium and water balance in the extracellular space, within the first week after admission of critically ill patients to the ICU. In addition, we found no relationship between the increase in sodium or water balance and direct measurement of blood volumes on D_2_ and D_7_. We found that MR-proADM thresholds of 0.865 nmol/L for ∆Na^+^ and 1.125 nmol/L for ∆H_2_0 were predictive of fluid and of salt overloads, respectively. Moreover, MR-proADM was related to the concurrent SOFA score.

Excessive sodium and fluid balance is a risk factor for morbidity and mortality in critically ill patients [7–9, 33]. A reliable and easy to measure surrogate biomarker could be very useful in improving the monitoring of fluid balance and identification of patients with a positive interstitial fluid and sodium balance. This biomarker should enable us to better personalize therapy and to guide fluid resuscitation and administration of vasopressors or diuretics.

While MR-proADM seems to be a particularly reliable indicator of sodium and fluid balance, it does not exclusively indicate capillary permeability. Indeed, MR-proADM is a stable fragment of pro-adrenomedullin which reflects levels of the rapidly degraded active peptide adrenomedullin [22]. In addition to its role in vascular endothelial barrier permeability in blood vessels [23], adrenomedullin also stabilizes the lymphatic endothelial barrier [34] and is implicated as an important pleiotropic effector of the host defense mechanism [35].

In the ICU, fluid balance is evaluated by the use of input/output charts and by the daily measure of weight. However, it is time consuming and difficult to perform accurately. In addition, the present study identified a weak relationship between weight and fluid balance. Measurement of patient’s weight in inflammatory disorders may be biased by practical issues but also by loss of muscle mass, interfering with the estimate of fluid overload. Moreover, we found that the usual markers of extracellular volume, such as plasma proteins and hemoglobin also had a weak relationship with sodium or water balance. Thus, the monitoring of sodium and fluid balance is currently a difficult task using tools that have their own limits. MR-proADM concentration, on the other hand, offers a measure of sodium balance and extracellular space, which could be used as a good surrogate to improve fluid balance monitoring. Possibly, it will be useful in the future to test a score centered on MR-proADM but also taking into account simple values such as basal weight or plasma proteins, to easily obtain an even better measurement.

No marker accurately estimated the TBV, PV or RBCV. Moreover, ∆Na^+^ or ∆H_2_O are not predictors of blood volume. We found no relationship between fluid balance and plasma volume. This was particularly unexpected, because plasma volume expansion is the main justification for fluid infusion. Yet, plasma was the only volume that was in the normal range on D_2_ and D_7_. The absence of correlation between PV and fluid balance supports the hypothesis of possible trapping of Na^+^ and water in the interstitial volume [36, 37]. Tighter control of PV would be useful in daily practice, though we did not find relationships with biomarkers, proteins or hemoglobin. This issue warrants further examination with a combination of other biomarkers or predictors.

The good correlation between the measurements of PV deducted from RBCV with ^51^Cr and directly measured with ^125^I-albumin at D_7_ strengthened our results. The distribution volume of albumin may be larger than that obtained with RBC, especially when the capillary permeability is pathologically increased. On D_7_ the difference was weak, suggesting that the capillary permeability was nearly repaired. While it would have been worthwhile to examine this comparison on D_2_, this was precluded by the interactions between ^125^I and some of the measurements of biomarkers.

The observation of a decreased RBCV is common and explains low TBV. The measurement of RBCV with ^51^Cr is a recognized method but requires time [28]. As hematocrit, hemoglobin is dependant of the ratio between RBCV and PV, we found, as others, that hemoglobin concentration is a poor surrogate for RBCV in ICU [38]. EPO is related to RBCV but the ROC curve is not sufficiently discriminative. We found no good biomarker of RBCV to help address this problem.

The present study has some limitations. First, we found no marker that describes blood volume. We need a better understanding of all physiologic determinants, including capillary permeability, hormonal influences and low RBCV and interactions among theses determinants. On the other hand, we identified a biomarker of cumulative salt and water balance, which could be an interesting tool for fluid management in the ICU. Second, we made our study measurements on D_2_, D_5_ and D_7_, long after the admission of patients into the ICU, often after the peak of the disease manifestation. The distribution of extracellular volume may be different at D_0_ during initial resuscitation. The precise timing of measurements may also be a critical factor. While our study observed the changes that took place on D_2_ and D_7_ after an acute event, further studies are needed to fill the gaps.

Our study was observational. Patients who were included reflect the daily practice of our service. However, the group of patients as a covariate did not influence the prediction of fluid balance or blood volume, suggesting that volume abnormalities are independent of the pathology. Other studies will need to confirm our results.

## Conclusion

A positive sodium balance is an important negative prognostic factor. We found that MR-proADM is a valuable surrogate to evaluate sodium and fluid overload in the first week after an acute and critical inflammatory illness. MR-proADM could improve the fluid balance monitoring and guide fluid resuscitation and administration of vasopressors or diuretics. After this first step, further studies are needed to test the ability of MR-proADM monitoring to control or prevent fluid overload and organ failure.

## Additional files

Additional file 1: Lack of relationship between cumulative sodium balance *(∆Na* ^*+*^, g) (A); cumulative fluid balance *(∆H*_*2*_ *O*, L) and total blood volume (*TBV*, mL/Kg), red blood cell volume (*RBCV*, mL/Kg) and plasmatic volume (*PV*, mL/Kg) at D_2_ and D_7_ (B). (DOCX 47 kb)
Additional file 2: Random forests perform regression using decision trees. It is a non parametric machine learning method using a series of random decisions at each tree. We used random forests to perform an initial choice between the large number of variables to enter in the second phase of regression. A SOFA (*top*), ∆H_2_O (*middle*) and ∆Na^+^ (*bottom*) variable importance measured by random forests. This figure displays the dot chart used to choose the variables entered into the linear mixed-effect regression model. On the *left* is the normalized mean square error (*MSE*), on the *right,* the node impurity is the residual sum of squares. B Results of the mixed effect modeling for ∆Na^+^, ∆H_2_O and SOFA using the lme procedure from R. (DOCX 322 kb)

## Abbreviations

^125^I-albumin: radioactive iodine
^51^Cr-RBC: radioactive chromium
ANP: atrial natriuretic peptide
AUC: area under the curve
BMI: body mass index
CI: confidence interval
EPO: erythropoietin
ICU: intensive care unit
IGS II: simplified index of gravity
IQR: interquartile range
ISS: injury severity score
MR-proADM: mid-regional pro-adrenomedullin
NCT: severe non-cerebral trauma
NYHA: New York Heart Association
PPS: postoperative peritonitis with shock
PV: plasma volume
RBCV: red blood cell volume
ROC: receiver operating characteristic
SAH: aneurysmal subarachnoid hemorrhage
SBT: severe brain trauma
SOFA: Sequential Organ Failure Assessment
TBV: total blood volume
WFNS: World Federation of Neurosurgical Societies
