# Supplementary material for: Mid-regional pro-adrenomedullin (MR-proADM), a marker of positive fluid balance in critically ill patients: results of the ENVOL study
Source: Crit Care. 2016 Nov 9;20:363. doi: 10.1186/s13054-016-1540-x (PMC5101658; doi:10.1186/s13054-016-1540-x)
Supplement: Additional file 1: — Lack of relationship between cumulative sodium balance (∆Na +, g) (A); cumulative fluid balance (∆H 2 O, L) and total blood volume (TBV, mL/Kg), red blood cell volume (RBCV, mL/Kg) and plasmatic volume (PV, mL/Kg) at D2 and D7 (B). (DOCX 47 kb) [file 13054_2016_1540_MOESM1_ESM.docx]

Additional file 1. Lack of relationship between A- cumulative sodium balance (∆Na^+^, g) ; B- cumulative fluid balance (∆H_2_O, L) and total blood volume (TBV, mL/Kg), red blood cells volume (RBCV, mL/Kg) and plasmatic volume (PV, mL/Kg) at D_2_ and D_7_

A.

B.
