# Supplementary material for: Mid-regional pro-adrenomedullin (MR-proADM), a marker of positive fluid balance in critically ill patients: results of the ENVOL study
Source: Crit Care. 2016 Nov 9;20:363. doi: 10.1186/s13054-016-1540-x (PMC5101658; doi:10.1186/s13054-016-1540-x)
Supplement: Additional file 2: — Random forests perform regression using decision trees. It is a non parametric machine learning method using a series of random decisions at each tree. We used random forests to perform an initial choice between the large number of variables to enter in the second phase of regression. A SOFA (top), ∆H2O (middle) and ∆Na+ (bottom) variable importance measured by random forests. This figure displays the dot chart used to choose the variables entered into the linear mixed-effect regression model. On the left is the normalized mean square error (MSE), on the right, the node impurity is the residual sum of squares. B Results of the mixed effect modeling for ∆Na+, ∆H2O and SOFA using the lme procedure from R. (DOCX 322 kb) [file 13054_2016_1540_MOESM2_ESM.docx]

Additional file 2.docx

Random forests perform regression using decision trees. It is a non parametric machine learning method using a series of random decision at each tree. We used random forests to perform an initial choice between the large number of variables to enter in the second phase of regression.

A- SOFA (top), ∆H_2_O (middle) and ∆Na^+^ (bottom) variable importance measured by Random Forests. This figure displays the dotchart used to choose the variables entering in the linear mixed effect regression model. On the left is the normalized Mean Square Error (MSE), on the right, the node impurity is the residual sum of squares.

B- Results of the mixed effect modeling for ∆Na^+^, ∆H_2_O and SOFA using the lme procedure from R:

SOFA

Value Std.Error DF t-value p-value

(Intercept) 3.665740 0.4096829 130 8.947750 0.0000

GNa 0.096543 0.0169470 130 5.696736 0.0000

MR.proADM 1.246843 0.2300886 130 5.418967 0.0000

EPO 0.005450 0.0025144 130 2.167580 0.0320

Marginal R^2^, i.e the proportion of variance explained by the fixed effects = 42 %

Conditional R^2^, i.e the proportion of variance explained by both the fixed and random effects = 83 %

∆H_2_O

Value Std.Error DF t-value p-value

(Intercept) 12.205226 1.7096953 131 7.138831 0e+00

MR.proADM 0.805913 0.1721113 131 4.682512 0e+00

Hb -0.570888 0.1290556 131 -4.423580 0e+00

Fluid.J0 0.293148 0.0808281 65 3.626805 6e-04

Prot -0.085276 0.0250226 131 -3.407942 9e-04

Marginal R^2^ = 36 %, conditional R^2^ = 93 %

∆Na^+^

Value Std.Error DF t-value p-value

(Intercept) 102.64668 16.996010 131 6.039457 0.0000

MR.proADM 3.97662 1.635130 131 2.431991 0.0164

Prot -1.97757 0.202270 131 -9.776884 0.0000

Poids 0.59489 0.149950 65 3.967235 0.0002

Angio 0.08556 0.039963 131 2.141132 0.0341

Marginal R^2^ = 48 %, conditional R^2^ = 89 %
